# Association between Dietary Vitamin A and HPV Infection in American Women: Data from NHANES 2003–2016

**DOI:** 10.1155/2020/4317610

**Published:** 2020-01-10

**Authors:** Xian Huang, Chi Chen, Fangfang Zhu, Yingxuan Zhang, Qiuting Feng, Jingwei Li, Qingying Yu, Yanlan Zhong, Songping Luo, Jie Gao

**Affiliations:** ^1^First School of Clinical Medicine, Guangzhou University of Chinese Medicine, # No. 12 Ji Chang Road, 510405, Guangzhou City, Guangdong Province, China; ^2^Department of Immunology and Microbiology, Guiyang College of Traditional Chinese Medicine, 84# Shi Dong Road, 550001 Guiyang, Guizhou, China; ^3^Department of Gynecology, # No. 16 Ji Chang Road, The First Affiliated Hospital of Guangzhou University of Traditional Chinese Medicine, 510405 Guangzhou City, Guangdong Province, China

## Abstract

**Objective:**

Evidence regarding the relationship between vitamin A and HPV infection was limited. Therefore, this study is designed to investigate whether vitamin A was independently related to HPV infection in 13412 American women from NHANES for seven cycles.

**Methods:**

The present study is a cross-sectional study. A total of 13412 eligible participants who had available HPV tests and vitamin A intake data were registered in the NHANE database from 2003 to 2016. The targeted independent variable and the dependent variable were vitamin A measured at baseline and HPV infection, respectively. We analyzed the association between dietary vitamin A intake and the prevalence of HPV infection. Besides, GAM and smooth curve fittings were used to address the nonlinear relationship between vitamin A and HPV infection to determine the effect of HPV infection.

**Results:**

The result of fully adjusted binary logistic regression showed vitamin A was not associated with the risk of HPV infection after adjusting confounders (odds ratio = 0.97, 95% confidence interval: 0.97–1.02). A nonlinear relationship was detected between vitamin A and HPV infection, whose inflection point was 10.5 of log2 vitamin A (by the recursive algorithm). One unit increase of log2 vitamin A is associated with the 10% reduced risk of HPV infection when dietary vitamin A is < 1448.155mcg. Conversely, when the dietary vitamin A intake is ≧1448.155 mcg, for each additional log2 of vitamin A, the risk of HPV infection increased by 70%.

**Conclusions:**

We found that dietary vitamin A was quite different from the trend of HPV infection in different confidence intervals. The results suggested that an appropriate amount (95% CI: 0.9–1.0, <10.5 of log2 transformer, i.e., 1448.155 mcg) of dietary vitamin A may be beneficial to prevent HPV infection. However, excessive intake of dietary vitamin A (95% CI: 1.1–2.8, ≧10.5 of log2 transformer, i.e., 1448.155 mcg) may increase the risk of HPV infection.

## 1. Introduction

Vitamin A, an essential fat-soluble vitamin throughout the human life cycle, is widely found in eggs, milk, liver, fresh vegetables, and fruits. It cannot be synthesized by itself, so it must be taken through diet [1]. The 2015 American Dietary Guidelines recommended that women consume 700 mcg of vitamin A daily [2]. However, the Dietary Guidelines Advisory Committee (DGAC) determined that even though vitamin A was an indispensable nutrient, many Americans tended to ignore. Their vitamin A consumption does not meet the estimated average demand (EAR) or adequate intake (AI) of the Institute's dietary reference intakes (DRIs) [3]. Evidence from previous studies suggested that insufficient intake of vitamin A can cause numerous diseases, for example, HPV infection, obesity, and visual impairment [4–6]. Therefore, the government tried to change the status of micronutrient deficiencies such as vitamin A through the enrichment and/or fortification (E/F) of food supply policy [7]. Vitamin A accounts for a large proportion of enrichment and/or fortification (E/F) of food supply; thus, potential harm due to excessive vitamin A intake should be taken into consideration [8]. For instance, excessive intake of vitamin A can cause liver damage, jaundice, and cirrhosis [9]. Therefore, inadequate or excessive intake of vitamin A may lead to adverse events.

Human papillomavirus (HPV) infection has become a major public health challenge for women. In the United States, HPV infection resulted in about 30,000 cases of cancer each year [10]. 70% of high-risk HPV infections are associated with cervical cancer, which is the fourth most common cancer affecting women worldwide [11]. Therefore, to prevent cervical cancer caused by HPV infection, many countries urgently started to study and inoculate the HPV vaccine to prevent it since 2006 [12]. However, with low coverage of HPV vaccination, it seems to be a most pressing question to find out how to prevent HPV infection in common means [13, 14].

Previous studies have confirmed that dietary factors, vitamin A, in particular, is closely related to decreased HPV-associated tumors (cervical cancer, squamous cell cancers of the head and neck, and prostate cancer) [15–18]. However, findings from former studies regarding the relationship between vitamin A and HPV infection were limited [19, 20]. Since the cancer risk of HPV infection has been affirmed, it is necessary to study the association between vitamin A and HPV infection from the perspective of preventing such tumors.

## 2. Methods

### 2.1. Data Source

The National Health and Nutrition Examination Survey (NHANES), a cross-sectional study from 1999 to the present, is designed to assess the health and nutritional status of adults and children in the United States. NHANES covers interviews and medical examinations with a focus on various health and nutrition measurements and is the main program of the National Center for Health Statistics (NCHS). More detailed content can be found on the NHANES official website (https://www.cdc.gov/nchs/nhanes/).

### 2.2. Study Population

We selected eligible common data files from the large-scale population-based NHANES database, an ongoing cross-sectional observational study for seven cycles. All participants signed the informed consent approved by the NCHS Research Review Board and received appropriate compensation.

**Figure 1:**
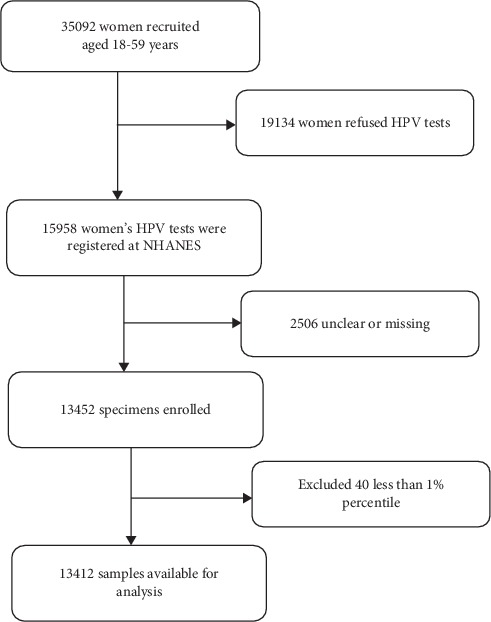
Flowchart of selection.

In 2003–2016, 35092 women aged 18 and 59 were registered in the NHANES database; among them, 19,134 women refused to undergo HPV testing. However, 2506 women's inspection results were unclear or missing; as a result, a total of 13452 specimens were included in our analysis. For vitamin A (log2 transformer), 40 people's survey results were less than 1% percentile, so we excluded this part of the population. In total, 13412 out of 35092 women were available for final analysis. More details of the selected sample can be seen in the below flowchart (Figure 1).

### 2.3. Variables

Vitamin A was obtained and recorded at baseline as a continuous variable. The public data files on dietary were obtained from the Centers for Disease Control and Prevention (CDC). The automated multiple-pass method (AMPM), a dietary interview computer program, was used to collect the participants' two nonconsecutive 24-hour dietary recalls [21]. Trained and skilled interviewers conducted the first recall by using an in-person interview at the Mobile Examination Center. The second recall was conducted by telephone interviews within 3–10 days [22, 23]. The acquisition and measurement of vitamin A can be found in the NHANES database (https://wwwn.cdc.gov/Nchs/Nhanes/2003-2004/DR1IFF_C.htm).

We described HPV infection as the following detailed process of measurement: (1) extract vaginal cells from the participants with vaginal wipes; (2) the processed samples were stored and sent to the Centers for Disease Control and Prevention, Atlanta, GA, for analysis; and (3) the vaginal swab specimen DNA was detected and analyzed by the Roche prototype line blot assay and the Roche Linear Array (LA) HPV Genotyping kit. More information on HPV measurements can be found on the website (https://wwwn.cdc.gov/Nchs/Nhanes/2003-2004/L37SWA_C.htm#LBDHPCR).

Based on published studies, the covariates used in our research can be classified as demographic data or variables that can affect vitamin A or HPV infection used in the NHANES database [24–26]. Hence, we used the following variables to construct the fully adjusted model: (1) continuous variable: age (years) and body mass index (BMI, kg/m^2^) (obtained at baseline) and (2) categorical variables: number of boyfriends have had in the past one year (times), race, education level, marital status, smoking more than 100 cigarettes in their lifetime, drink at least 12 glasses of alcohol/1 year, and vaginal or anal intercourse times in the past year (times) (obtained at baseline).

### 2.4. Statistical Analysis

In our study, if the continuous variable was normally distributed, it was presented as mean ± standard, otherwise as medium (min, max). Furthermore, we expressed categorical variables as a percentage. The distribution of vitamin A in the population was skewed, so we made a conversion of log2. Our statistical analyses consisted of three main steps. First of all, we employed multivariate binary logistic regression by constructing three models: model 1, no covariates adjusted; model 2, merely adjusted sociodemographic data; and model 3, explored the nonlinearity of vitamin A and HPV infection, and a generalized additive model and smooth curve fitting (penalized spline method) were employed. In Step 2, we detected the relationship of nonlinearity between vitamin A and HPV infection, and the inflection point employing a recursive algorithm was calculated. In Step 3, we constructed a two-piecewise binary logistic regression on both sides of the inflection point. The best-fit model based on the *P* values for the log-likelihood ratio test was determined.

The whole data analyses were employed with the statistical software packages R (http://www.R-project.org, The R Foundation) and EmpowerStats (http://www.empowerstats.com, X&Y Solutions, Inc., Boston, MA). We determined that *P* values less than 0.05 (two-sided) were statistically significant.

## 3. Results

### 3.1. Baseline Characteristics of Selected Participants

A total of 13412 participants were involved in this research before we screened participants according to inclusion and exclusion criteria as presented in Figure 1. The baseline characteristics of selected women are presented in Table 1 according to HPV infection (dichotomous variable).

### 3.2. Results of Unadjusted and Adjusted Binary Logistic Regression

In this research, we constructed three main models to explore the independent effects of vitamin A on HPV infection by univariate and multivariate binary logistic regression. The effect sizes (OR), 95% confidence intervals, and *P* value are listed in Table 2. We found the intake of dietary vitamin A was negatively associated with the risk of HPV infection in the unadjusted model (model 1). The model-based effect size can be explained as each additional increase in log2 transformer vitamin A consumption was associated with a 10% lower risk of HPV infection (OR: 0.90, 95% CI: 0.87–0.92).

In the fully adjusted model (OR: 0.97, 95% CI: 0.92–1.02) and the GAM (adjusted all covariates presented in Table 1) (OR: 0.97, 95% CI: 0.92–1.02), there was no statistical difference between vitamin A and HPV infection, even though vitamin A was converted from a continuous variable to a categorical variable (quartile of vitamin A) for sensitivity analysis. As the relationship between vitamin A and HPV infection is nonlinear, the GAM permits nonlinear and linear relationships to coexist in our model in this research. Moreover, we allowed the covariates (age, BMI, and poverty income ratio) into the equation as a curve, but surprisingly, the results were the same (OR: 0.97, 95% CI: 0.92–1.02). Besides, we found the trend of the effect size in different vitamin A groups was nonequidistant.

All covariates listed in Table 1 were adjusted in the fully adjusted model. In the GAM model, all covariates listed in Table 1 were adjusted. However, the age, BMI, and poverty income ratio were adjusted as nonlinearity.

### 3.3. Results of Nonlinearity of Vitamin A and HPV Infection

**Figure 2:**
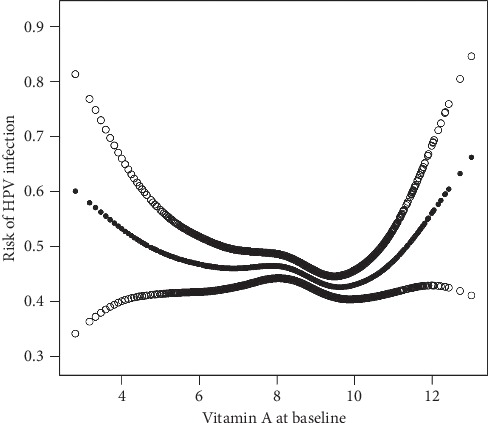
Nonlinear relationship between vitamin A and HPV infection.

In our present study, we aimed at analyzing the nonlinear relationship between vitamin A and HPV infection (Table 3 and Figure 2). We found that the relationship between vitamin A and HPV infection was nonlinear after adjusting age (years), body mass index (BMI, kg/m^2^), race, and other covariates by smooth curve and the effects of the generalized additive model. Both binary logistic regression and two-piecewise binary logistic regression were used to fit the relationship and choose the best-fit model based on *P* for log-likelihood ratio test.

Two-piecewise binary logistic regression for fitting the association between vitamin A and HPV infection was selected because it can accurately represent the relationship. By using two-piecewise binary logistic regression and recursive algorithm, the inflection point was calculated as 10.5. The effect value and 95% CI were, respectively, 0.9 and 0.9–1.0 on the left side of an inflection point. Identically, on the right side of the inflection point, the effect size and 95% CI were 1.7 and 1.1–2.8, respectively.

The covariate-adjusted in the two models was the same as the GAM presented in Table 2.

## 4. Discussion

In summary, we found that vitamin A (log2 transformer) was not associated with HPV infection after adjusting other covariates (OR: 0.97, 95% CI: 0.92–1.02) in the binary logistic regression equation. It appeared that there was a possibility of a nonlinear relationship by turning vitamin A into a categorical variable. Hence, we further considered the nonlinear relationship and found that there was a curvilinear relationship between vitamin A and HPV infection. Furthermore, our findings indicated that the tendency of the effect sizes on both the left and right sides of the inflection point was not consistent (left OR: 0.9, 95% CI (0.9, 1.0); right OR: 1.7, 95% CI (1.1, 2.8)). This above result suggested an approximately U-shaped curve on the independent association between vitamin A and HPV infection.

Shannon et al. performed a case-control study including 50 women with in situ cervical cancer and 125 controls, and 134 invasive cervical cancer cases and 384 controls. They believed that dietary vitamin A intake was found to be related to a reduced risk of HPV infection-associated in situ and invasive cervical cancer [27]. Lehtinen et al. performed a nested case-control study of 38 invasive cervical cancer cases and 116 controls and found that low level of serum vitamin A (retinol) was associated with HPV infection [28]. Their conclusions were partially consistent with our findings that there was a negative correlation between vitamin A and HPV infection. However, unlike them, we found that this negative correlation exists only when the dietary vitamin A intake is <10.5 (log2 transformer). We also have a new finding based on them, that is, when the dietary vitamin A intake is ≧10.5 (log2 transformer), i.e., 1448.155 mcg, vitamin A is positively related to HPV infection. Some studies suggested that dietary vitamin A was not associated with HPV infection [29–32]; their results are inconsistent with the findings of this study. We speculated that the causes of the distinct findings may be as follows: (1) population varies in different research studies. These studies, which were inconsistent with our findings, were targeted at the female, in different countries, who were infected with HPV; (2) their conclusions did not clarify the nonlinear relationship or curve relationship; and (3) compared with our work, the sample sizes of these studies were slightly smaller.

We consider the clinical value as the following points: (1) To our best knowledge, it is the first time to observe the U-shaped relationship between vitamin A and HPV infection in American women in a large cross-sectional study. (2) The findings of our research should be helpful to develop dietary guidelines and prevent strategies for HPV infection. (3) With reference to the NHANES database, we have now enrolled more than 3,000 women to conduct a community-based cohort study (Ling Nan cohort), which aims at exploring the association between HPV infection and dietary, psychological, and work factors in Chinese women.

Our research has the following advantages. Our sample size is relatively large compared with previous similar studies. To ensure the robustness of data analysis and explore the true relationship between vitamin A and HPV infection, we did a series of sensitivity analyses and fully explained the nonlinear relationship. Our study is an observational study, which is susceptible to potential confounding. Therefore, strict statistical adjustments were used to minimize residual confounders. Furthermore, few studies have elucidated the U-shaped relationship of vitamin A and HPV infection in recent years.

However, there are some limitations to our study. Firstly, our study subjects are 13412 American women aged 18–59 years. Hence, the study cannot be applied to men or people beyond the age range. Secondly, we cannot get a causal relationship between vitamin A and HPV infection because our research is a cross-sectional study. Thirdly, dietary vitamin A is obtained from NHANES participants' 24-hour dietary recall interviews. Thus, like other studies, our research has inherent and unavoidable weaknesses and may have recall bias, measurement bias, and so on. However, the directionality (larger or smaller) caused by recall bias and measurement bias was fair for each participant, and it did not interfere with our results. Last but not least, our study only discussed the relationship between dietary vitamin A and HPV infection, but did not discuss serum levels of vitamin A (retinol or retinoic acid) and HPV infection. Therefore, the comparison between serum vitamin A and dietary vitamin A with HPV infection should be paid attention in the future.

## Figures and Tables

**Table 1:** Baseline characteristics of selected participants.

Exposure	No HPV infection	HPV infection	*P* value
BMI, mean ± SD (Kg/m^2^)	28.89 ± 7.68	29.06 ± 7.71	0.292
Age, mean ± SD (years)	38.21 ± 12.08	35.86 ± 12.22	<0.001
Poverty income ratio, mean ± SD	2.77 ± 1.69	2.27 ± 1.65	<0.001
How many boyfriends have had in the past one year, mean ± SD (times)	1.10 ± 2.53	1.83 ± 5.03	<0.001
Race (%)			<0.001
Mexican American	19.88	16.92	
Other Hispanic	8.48	9.37	
Non-Hispanic white	45.78	38.90	
Non-Hispanic black	14.70	28.34	
Other races	11.16	6.47	
Education level (%)			<0.001
Less than 9th grade	7.17	6.13	
9–11th grade	10.94	14.26	
High school grade	17.30	21.79	
College graduate or above	32.18	36.39	
Some college or AA degree	32.41	21.43	
Marital status (%)			<0.001
Married or cohabiting	68.61	46.88	
Widowed or divorced or separated	31.39	53.12	
Smoking more than 100 lifetimes (%)			<0.001
None	70.95	58.25	
Yes	29.05	41.75	
Drink at least 12 glasses of alcohol/1 year (%)			<0.001
0	37.37	29.32	
1	62.63	70.68	
Vaginal or anal intercourse times in the past year (times) (%)			<0.001
1	3.90	2.94	
2	3.23	4.03	
12–51	20.96	25.14	
52–103	36.66	32.79	
104–364	21.63	20.06	
≥365	12.71	13.63	
0	0.91	1.40	

**Table 2:** Multivariate analysis for the linear relationship between vitamin A and HPV infection.

Exposure	Nonadjusted model (OR (95% CI), *P* value)	Fully adjusted model (OR (95% CI), *P* value)	GAM (OR (95% CI), *P* value)
Vitamin A (log2 transformer, mcg)	0.90 (0.87, 0.92), <0.0001	0.97 (0.92, 1.02), 0.2002	0.97 (0.92, 1.02), 0.2017
Q1 (2.80–8.06)	Ref	Ref	Ref
Q2 (8.06–8.87)	0.91 (0.83, 1.00), 0.0503	1.08 (0.91, 1.28), 0.4009	1.08 (0.91, 1.28), 0.3999
Q3 (8.87–9.54)	0.75 (0.68, 0.83), <0.0001	0.86 (0.72, 1.03), 0.0950	0.86 (0.72, 1.03), 0.0944
Q4 (9.54–14.83)	0.72 (0.65, 0.79), <0.0001	0.96 (0.80, 1.14), 0.6173	0.96 (0.80, 1.14), 0.6212
*P* for trend	<0.0001	0.2193	0.2207

Nonadjusted model adjusted for none.

**Table 3:** Nonlinearity addressing by a weighted two-piecewise linear model.

Outcome:	HPV infection (OR (95% CI), *P* value)
Fitting by standard linear model	
Fitting by two-piecewise linear model	1.0 (0.9, 1.0), 0.199
Inflection point (K)	10.5
<10.5	0.9 (0.9, 1.0), 0.035
≧10.5	1.7 (1.1, 2.8), 0.018
Log-likelihood ratio	0.011

## Data Availability

The data used to support the findings of this study were supplied by the NHANES public database (https://www.cdc.gov/nchs/nhanes/).
